# Glucose-6-phosphate Reduces Fosfomycin Activity Against *Stenotrophomonas maltophilia*

**DOI:** 10.3389/fmicb.2022.863635

**Published:** 2022-05-10

**Authors:** Teresa Gil-Gil, José Luis Martínez

**Affiliations:** ^1^Centro Nacional de Biotecnología, CSIC, Madrid, Spain; ^2^Programa de Doctorado en Biociencias Moleculares, Universidad Autónoma de Madri, Madrid, Spain

**Keywords:** fosfomycin, *Stenotrophomonas maltophilia*, adjuvant, intrinsic resistance, glucose-6-phosphate

## Abstract

It is generally accepted that fosfomycin activity is higher in the presence of glucose-6-phosphate, since its inducible transporter UhpT is one of the gates for fosfomycin entry. Accordingly, fosfomycin susceptibility tests are performed in the presence of this sugar; however, since *Stenotrophomonas maltophilia* lacks UhpT, it is doubtful that glucose-6-phosphate might be a fosfomycin adjuvant in this microorganism. The aim of the work was to determine whether glucose-6-phosphate or other metabolites may alter the activity of fosfomycin against *S. maltophilia*. To that goal, checkerboard assays were performed to analyze the synergy and antagonism of compounds, such as glucose-6-phosphate, fructose, phosphoenolpyruvate, and glyceraldehyde-3-phosphate, among others, with fosfomycin. Besides, minimal inhibitory concentrations of fosfomycin against a set of clinical *S. maltophilia* isolates presenting different levels of expression of the SmeDEF efflux pump were determined in the presence and absence of said compounds. Finally, intracellular fosfomycin concentrations were determined using a bioassay. Our results show that, opposite to what has been described for other bacteria, glucose-6-phosphate does not increase fosfomycin activity against *S. maltophilia*; it is a fosfomycin antagonist. However, other metabolites such as fructose, phosphoenolpyruvate and glyceraldehyde-3-phosphate, increase fosfomycin activity. Consistent with these results, glucose-6-phosphate decreases fosfomycin internalization (a feature against current ideas in the field), while the other three compounds increase the intracellular concentration of this antibiotic. These results support that current standard fosfomycin susceptibility tests made in the presence of glucose-6-phosphate do not account for the actual susceptibility to this antibiotic of some bacteria, such as *S. maltophilia*. Finally, the innocuous metabolites that increase *S. maltophilia* susceptibility to fosfomycin found in this work are potential adjuvants, which might be included in fosfomycin formulations used for treating infections by this resistant pathogen.

## Introduction

The increased drug resistance in Gram-negative bacilli (GNB), together with the lack of new antibiotics, has renewed the interest in less-used antibiotics, such as fosfomycin (Behera et al., 2018). Fosfomycin is analogous to phosphoenolpyruvate (PEP), the substrate of MurA (UDP-N-acetylglucosamine enolpyruvyl transferase). MurA catalyzes the first step in peptidoglycan biosynthesis, the transfer of enolpyruvate from PEP to uridine 5'-diphosphate-N-acetylglucosamine (UDP-GlcNAc). Fosfomycin covalently binds to a cysteine residue in the active site of MurA, the only known fosfomycin target, inactivating it. MurA inactivation produces the accumulation of peptidoglycan precursor monomers inside the cell, peptidoglycan cannot be synthesized, and bacterial cell lysis and death happen (Raz, 2012; Behera et al., 2018).

One GNB pathogen characterized by its low susceptibility to commonly used antibiotics is *Stenotrophomonas maltophilia* (Sanchez et al., 2009; Brooke, 2012; Brooke et al., 2017; Gil-Gil et al., 2020b; Sanz-García et al., 2021), which is considered to be intrinsically resistant to fosfomycin. Consequently, even though therapeutic options for treating *S. maltophilia* infections are scarce, fosfomycin is not considered as a valid alternative (Lu et al., 2011; Rizek et al., 2015; Falagas et al., 2016).

It is generally accepted that the gates for fosfomycin entry are the transporters of sugar the phosphates hexose phosphate (UhpT) and glycerol-3-phosphate (GlpT) (Tsuruoka and Yamada, 1975). Their expression is under metabolic control; when the nutritional bacterial status favors the use of these sugar phosphates, the expression of their transporters is triggered, and fosfomycin activity increases (Scortti et al., 2018). Since *uhpT* expression is increased in different organisms by glucose-6-phosphate—and despite early findings showed that, for some bacteria, such as *Serratia marcescens, Pseudomonas aeruginosa*, and enterococci, glucose-6-phosphate does not clearly improve fosfomycin activity (Grimm, 1979)—it is generally accepted that fosfomycin has much greater activity when it is provided with this sugar (Martin-Gutierrez et al., 2020). Due to this fact, current microbiological tests to determine fosfomycin resistance/susceptibility include glucose-6-phosphate, although it has been reported that different bacterial species do not present such inducible transporters (Winkler, 1973). All these facts make cumbersome this generalization. Indeed, neither UhpT nor GlpT have been found in available *S. maltophilia* genomes (Gil-Gil et al., 2020a), suggesting that another transporter could be used by fosfomycin to enter in this species.

UhpT and GlpT inactivating mutations are the main mechanisms of fosfomycin resistance in different bacterial species (Blair et al., 2015). Despite this fact, *S. maltophilia* fosfomycin low-susceptibility mutants have been selected and they do not harbor mutations in putative fosfomycin transporters different than those previously reported in other organisms. These mutants present mutations that inactivate a set of glycolytic enzymes belonging to the Embden–Meyerhof–Parnas metabolic pathway (Gil-Gil et al., 2020a). Given the predicted absence of known sugar-phosphate transporters in the *S. maltophilia* genome and the implication of central carbon metabolic enzymes in fosfomycin resistance, we measured the effect of glucose-6-phosphate and other metabolites on *S. maltophilia* fosfomycin susceptibility. Opposite to current concepts, glucose-6-phosphate does not improve the activity of the antibiotic but it is antagonistic. However, other sugars improve such activity in *S. maltophilia*.

## Methods

### Growing Conditions and Susceptibility Testing

The strains used in the work are described in Table 1. Minimal inhibitory concentration (MIC) values were obtained on liquid media, namely lysogeny broth (LB) Lennox (Atlas, 1993) (Condalab), Mueller–Hinton (MH) (Atlas, 1993) (Condalab), sterile human urine, and synthetic cystic fibrosis sputum medium (SCFM) prepared as described below, by double dilution after 48 h at 37°C. To have intermediate antibiotic concentration values that allow better discrimination of small MIC differences, different assays, each one beginning with a different concentration of antibiotic, were performed for each condition. As suggested in Martinez et al. (2015), this allows obtaining a more precise value of the MIC in each case. In all cases, MICs were determined three times on different days and the results were always consistent. Urine has been obtained by pooling urine from six human volunteers, who did not receive antibiotic treatment last year. Urine was filtered through 0.2-μm-pore-size filters (Whatman) and conserved at −20°C, and the same batch was used for all experiments. For SCFM, amino acids were added from 100 mM stocks to a buffered base (6.5 mL 0.2 M NaH2PO4, 6.25 mL 0.2 M Na2HPO4, 0.348 mL 1 M KNO3, 0.122 g NH4Cl, 1.114 g KCl, 3.03 g NaCl, 10 mM MOPS, and 779.6 mL deionized water) in the following volumes: 8.27 mL L-aspartate, 10.72 mL L-threonine, 14.46 mL L-serine, 15.49 mL L-glutamate, 16.61 mL L-proline, 12.03 mL L-glycine, 17.8 mL L-alanine, 1.6 mL L-cysteine, 11.17 mL L-valine, 6.33 mL L-methionine, 11.2 mL L-isoleucine, 16.09 mL L-leucine, 8.02 mL L-tyrosine, 5.3 mL L-phenylalanine, 6.76 mL L-ornithine, 21.28 mL L-lysine, 5.19 mL L-histidine, 0.13 mL L-tryptophan, and 3.06 mL L-arginine. SCFM was adjusted to pH 6.8 and filtered through a 0.2-μm-pore-size filter. After sterilization, the following sterile components were added per liter: 1.754 mL 1 M CaCl2, 0.606 mL 1 M MgCl2, 1 mL 3.6 mM FeSO4·7H2O, 3 mL 1 M D-glucose, and 9.3 mL 1 M L-lactate (Palmer et al., 2007). Potential fosfomycin adjuvants were added when needed. MICs of ciprofloxacin and trimethoprim/sulfamethoxazole (SXT) were determined using MIC Test Strips (Liofilchem^®^) on Mueller–Hinton agar (MHA, Pronadisa) at 37°C, following supplier's instructions.

**Table 1:** Susceptibility of *S. maltophilia, E. coli*, and *P. aeruginosa* to antibiotics in the presence of potential fosfomycin adjuvants.

**Isolate**	**Origin**	**SmeDEF overexpression**	MICs (μg/mL)^*^						
			**Fosfomycin MICs in the presence of different compounds**					**Ciprofloxacin**	**SXT**
			**None**	**Glucose-6-phosphate (25 μg/mL)**	**Fructose (125 μg/mL)**	**PEP (200 μg/mL)**	**Glyceraldehyde-3-phosphate (85 μg/mL)**		
D457	Bronchial aspirate	No	256	512	128	128	128	0.75	1.125
F227	Blood	No	128	512	128	64	128	0.3	1.25
G51	Blood	No	64	128	64	32	32	0.38	0.44
F375	Blood	Yes	256	512	128	64	128	8	>32
E539	Pus from a wound	No	64	128	64	32	64	0.75	0.44
E999	Respiratory secretion	No	64	128	32	32	64	0.75	0.88
F861	Sputum	No	64	128	32	16	32	0.25	1.5
E729	Urine	Yes	128	256	128	64	64	>32	2
E301	Urine	No	128	256	64	64	64	0.5	0.5
D388	Urine	No	64	128	32	32	32	0.38	0.5
C048	Urine	No	128	256	64	64	64	0.63	0.88
C357	Urine	Yes	128	512	128	64	128	>32	3
*E. coli* K12	Lab collection	–	1.5	0.25	2	1.12	1.17	–	–
*P. aeruginosa* PA14	Lab collection	–	20	16	14	16	16	–	–

* *MICs were determined at least three times on different days, and the results were always the same. To have intermediate MIC values, different determinations, each one beginning with a different concentration of antibiotic, were performed for each condition*.

### Checkerboard Assay

For searching potential fosfomycin adjuvants, 96-well microtiter plates containing MH medium with 11 serial concentrations of fosfomycin in combination with each tested metabolite were inoculated with *S. maltophilia* D457 cultures containing around 10^3^ cells per well. The compounds added to the plates were D-glucose-6-phosphate, D-fructose, phospho(enol)pyruvic acid, DL-glyceraldehyde-3-phosphate, DL-glycerol-3-phosphate, L-alanine, L-proline, D-glucose, sodium succinate, DL-lactate, and UDP-GlcNAc; all of them were purchased from Sigma–Aldrich. After 48 h of incubation at 37°C without shaking, the growth was measured with a Spark 10M plate reader (Tecan) at OD 600 nm. The fraction inhibitory concentration (FIC) index (FICI) was calculated using standard methods as described (Eickhoff, 1969). An FIC index value of ≤ 0.5 was considered to indicate synergy, and an FIC index of ≥ 2 was considered to indicate antagonism.

### Determination of Intracellular Fosfomycin Concentrations

The amount of intracellular fosfomycin was measured using a bioassay as described (Zykov et al., 2018; Gil-Gil et al., 2020a; Laborda et al., 2021). Briefly, *S. maltophilia* D457 was grown until the exponential phase, and cells were pelleted by centrifugation at 4,500 × g for 3 min and resuspended in 1/20 of the culture volume under the same growing conditions. Then, fosfomycin was added at 2 mg/mL and bacteria were incubated for 60 min at 37°C. At that time, the cells were resuspended in 0.6 mL of 0.85% NaCl, and sequential dilutions of the suspensions were plated onto LB agar to determine the number of colony forming units (CFUs) per milliliter. It was determined that the fosfomycin concentration used does not impair *S. maltophilia* growth during the time of the assay, in agreement with previous studies (Gil-Gil et al., 2020a). In parallel cultures were centrifuged as described above and bacterial pellets were washed three times with 1 mL buffer containing 10 mM Tris (pH 7.3), 0.5 mM MgCl_2_, and 150 mM NaCl to remove the remaining fosfomycin. These bacterial suspensions were boiled at 100°C for 5 min and centrifuged at 11,900 × g for 10 min for removing cell debris. Sterilized cellulose disks were impregnated with 40 μL of each supernatant and deposited onto LB agar plates seeded with *E. coli* DH5α. The halos of inhibition were recorded after 20 h of growth at 37°C and compared with those of disks containing different amounts of fosfomycin, from 0.625 to 10 μg, that were used to draw a standard curve.

The number of *S. maltophilia* cells used for each assay was estimated by sequential dilutions of the cultures as above mentioned (Gil-Gil et al., 2020a). Data were normalized to μg fosfomycin for 10^7^ cells.

Controls of lack of fosfomycin degradation upon boiling and sterility of the boiled extracts were performed as described (Gil-Gil et al., 2020a). In addition, controls using two derivatives from *P. aeruginosa* PA14, one lacking the fosfomycin transporter GlpT (control of low accumulation) and another lacking the fosfomycin inactivating enzyme FosA (that should accumulate more intracellular fosfomycin), were included in the assay. Assays were performed in triplicate, and the statistical significance of the differences in fosfomycin accumulation was estimated using an unpaired two-tailed *t*-test.

## Results and Discussion

It has been stated that strains of *Escherichia, Citrobacter, Enterobacter*, or *Klebsiella* species are more susceptible to fosfomycin when glucose-6-phosphate is added to the test medium (Barry and Fuchs, 1991), leading to the generalization of the concept that glucose-6-phosphate universally increases fosfomycin activity. Besides, early experiments suggest that glucose-6-phosphate can be present in human tissues at concentrations high enough to increase fosfomycin activity against organisms, such as *Escherichia coli, Klebsiella* spp., and *Proteus mirabilis* (Detter et al., 1983). Consequently, the Clinical and Laboratory Standards Institute and the European Committee on Antimicrobial Susceptibility Testing indications state that MICs of fosfomycin should be determined in the presence of 25 μg/mL glucose-6-phosphate (Wayne, 2020). However, later studies showed that in half of the tested strains, the effect of glucose-6-phosphate was very minor, particularly in organisms such as *P. aeruginosa, Proteus* spp., and *Serratia marcescens* (Greenwood et al., 1986). Further, concentrations of this sugar in human extracellular locations, such as urine, are much lower than those used in susceptibility tests, to the point that some fosfomycin-resistant mutants causing infections in these locations are only detectable in the absence of glucose-6-phosphate (Martin-Gutierrez et al., 2020). These features cast doubts on the reliability of the general use of glucose-6-phosphate in fosfomycin susceptibility tests to estimate *in vivo* fosfomycin activity, in particular for organisms lacking inducible glucose-6-phosphate transporters (Winkler, 1973) or in body locations where glucose-6-phosphate concentrations are likely to be low (Martin-Gutierrez et al., 2020).

Fosfomycin use is not indicated for the treatment of infections caused by *S. maltophilia* since this organism is considered as intrinsically resistant to this antibiotic. Note that this classification is based on current standardized protocols that, as stated above, include glucose-6-phosphate in MIC determinations. Notably, as indicated above, *S. maltophilia* genome does not encode either UhpT or GlpT homologs, the canonical fosfomycin transporters (Gil-Gil et al., 2020a), whose expression is triggered in different organisms in the presence of the sugar phosphates they transport. If these transporters are absent, it might be possible that glucose-6-phosphate does not improve fosfomycin activity in *S. maltophilia*. To address this possibility, fosfomycin susceptibility of the model strain D457 and a set of *S. maltophilia* clinical isolates (Alonso and Martinez, 2001) was determined in the absence and presence of glucose-6-phosphate. MICs were repeated three times on different days, and the results were always the same. Opposite to the information used for current official recommendations of susceptibility testing, glucose-6-phosphate does not increase antibiotic activity but displays the opposite effect; fosfomycin MICs are higher, not lower, in the presence of this supposed fosfomycin adjuvant (Table 1). The fact that, under the same experimental conditions, glucose-6-phosphate increases, as reported, the activity of fosfomycin against *E. coli*, while the effect was low in the case of *P. aeruginosa* (Grimm, 1979), supports the reliability of our assay (Table 1). The same result was obtained when MICs were determined in other media, such as urine or SCFM, that are closer to infective conditions (Table 2).

**Table 2:** Effect of different growing conditions in the susceptibility of *S. maltophilia* D457 to fosfomycin in the presence of different metabolites.

**Metabolite**	Fosfomycin MICs (μg/mL)^*^			
	**LB**	**MH**	**Urine**	**SCFM**
None	128	256	512	256
Glucose-6-phosphate (25 μg/mL)	512	512	1,024	512
Fructose (125 μg/mL)	32	128	256	128
PEP (200 μg/mL)	24	128	256	128
Glyceraldehyde-3-phosphate (85 μg/mL)	64	128	256	128

* *MICs were determined at least three times on different days, and the results were always the same. To have intermediate MIC values, different determinations, each one beginning with a different concentration of antibiotic, were performed for each condition*.

As mentioned, the inactivation of different metabolic enzymes further reduces *S. maltophilia* fosfomycin susceptibility, indicating that fosfomycin activity depends on *S. maltophilia* metabolism. It is then possible that other metabolites, besides glucose-6-phosphate, may alter *S. maltophilia* fosfomycin susceptibility. To address this possibility, checkerboard assays were performed with different metabolites, namely glucose, lactate, glucose-6-phosphate, glycerol-3-phosphate, succinate, fructose, alanine, proline, UDP-GlcNAc, PEP, and glyceraldehyde-3-phosphate. These compounds were chosen either because they are directly related to the fosfomycin activity or form part of specific carbon metabolic pathways, including those pathways whose inactivation has been reported to lead to reduced fosfomycin susceptibility (Gil-Gil et al., 2020a; Gil-Gil and Martínez, 2022). Besides, amino acids, such as proline and alanine, are the carbon sources present in a lung of a cystic fibrosis patient (Palmer et al., 2005, 2007). Finally, fructose was added first because PEP acts as a phosphate donor in fructose entrance and phosphorylation through the fructose phosphotransferase system (PTS) and second because fructose PTS expression changes in the presence of fosfomycin, PEP, and glyceraldehyde-3-phosphate (Gil-Gil et al., 2021). Moreover, the said work has shown that the effect of PEP and glyceraldehyde-3-phosphate on the *S. maltophilia* transcriptome is similar to that due to fosfomycin activity (Gil-Gil et al., 2021), further highlighting the close relationship between fosfomycin activity and carbon metabolism in *S. maltophilia*.

Noteworthy, glucose-6-phosphate, glycerol-3-phosphate, alanine, and proline showed an antagonistic effect with fosfomycin with FICI values of 2.36, 16.25, 2.23, and 2.02, respectively, further confirming that the substrates of the canonically proposed fosfomycin transporters, namely glucose-6-phosphate and glycerol-3-phosphate, unlike what happens in other bacterial species, significantly increase the resistance to this antibiotic.

**Figure 1:**
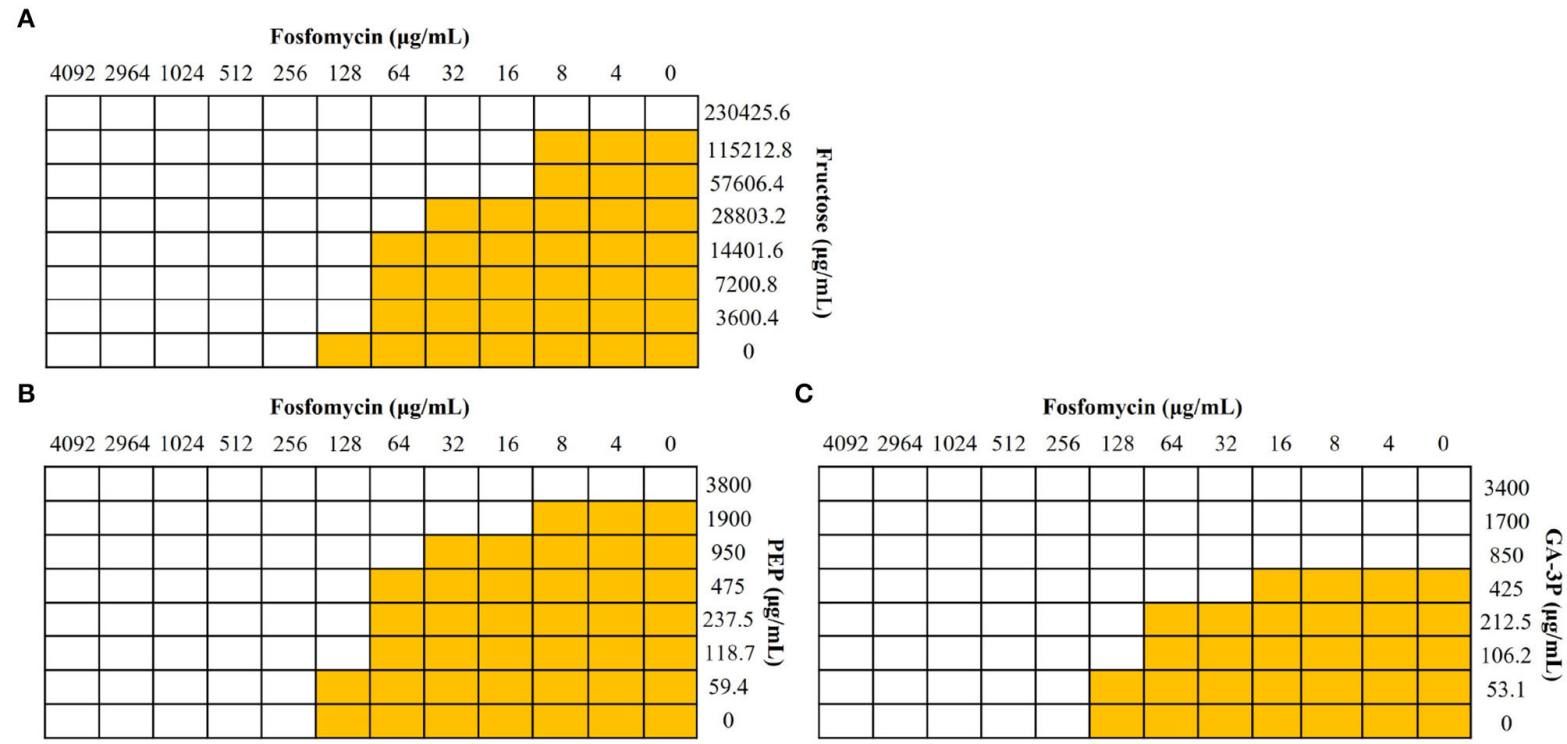
Effect of different metabolites in the activity of fosfomycin against *S. maltophilia*. Checkerboard analyses were performed with the *S. maltophilia* D457 strain. Wells with bacterial growth are represented in yellow, and wells in which there was no growth are represented in white. **(A)** Fructose showed a synergistic effect (FICI value of 0.31). **(B)** PEP and **(C)** glyceraldehyde-3-phosphate (GA-3P), despite not having a synergistic effect, FICI values of 0.56 and 0.62, respectively, showed an additive effect and improved fosfomycin activity against *S. maltophilia*.

Fosfomycin inhibits the action of MurA because it is structurally similar to PEP, one of the substrates of this enzyme and the other substrate being UDP-GlcNAc. Accordingly, competition for the covalent binding to MurA between these two metabolites and fosfomycin, when present at the same time, might result in a reduced binding of the antibiotic to its target and hence an increased resistance to fosfomycin. However, these compounds were not found to be fosfomycin antagonists, ruling out this hypothesis. Neither an antagonistic nor a synergistic effect was observed for glucose, succinate, lactate, and UDP-GlcNAc with FICI values of 0.63, 1, 0.54, and >0.5, respectively. UDP-GlcNAc solubility was not enough to firmly determine whether it may have synergistic or additive effects, being the highest UDP-GlcNAc concentration tested of 30 mg/mL. A synergistic effect was observed for fructose with a FICI value of 0.31. Despite the fact that a synergistic effect was not observed for PEP, a FICI value of 0.56, or glyceraldehyde-3-phosphate, a FICI value of 0.62, both compounds have an additive effect and improve fosfomycin activity (Figure 1) because of their inhibitory effect against *S. maltophilia* (Gil-Gil et al., 2021). When considering their potential therapeutic applicability, it is important noticing that there are no indications suggesting that fructose, PEP, or glyceraldehyde-3-phosphate could have a toxic effect in humans.

To further analyze the possible use of these compounds for improving fosfomycin activity, fosfomycin MICs of *S. maltophilia* D457 were obtained in the presence of different concentrations of fructose, PEP, or glyceraldehyde-3-phosphate that did not have a deleterious effect on bacterial growth (data not shown). The lowest concentrations that produced fosfomycin MIC changes of at least 2-fold−125 μg/mL fructose, 200 μg/mL PEP, and 85 μg/mL glyceraldehyde-3-phosphate—were chosen for further study. As shown in Table 2, the addition of either fructose, PEP, or glyceraldehyde-3-phosphate, at these concentrations, reduces fosfomycin MICs of the D457 model strain by at least 4-fold when compared with the standard conditions in the presence of glucose-6-phosphate in all tested media.

Quinolones and SXT, and more recently tigecycline, are among the few first-line antimicrobials that can be used for treating *S. maltophilia* infections (Biagi et al., 2020). Overexpression of the SmeDEF efflux pump produces cross-resistance to all of them (Alonso and Martinez, 2000; Garcia-Leon et al., 2014; Sanchez and Martinez, 2015; Blanco et al., 2019). To ascertain whether the effect of fructose, PEP, and glyceraldehyde-3-phosphate on fosfomycin susceptibility could be generalized, fosfomycin susceptibility in the presence of these compounds was determined in a set of clinical *S. maltophilia* isolates that includes antibiotic-resistant ones, some of them overexpressing SmeDEF and hence presenting a multidrug-resistant phenotype. In all cases, the results were consistent with a synergistic effect of the tested compounds with fosfomycin (Table 1), a feature that supports that this effect is independent on the level of SmeDEF expression.

**Figure 2:**
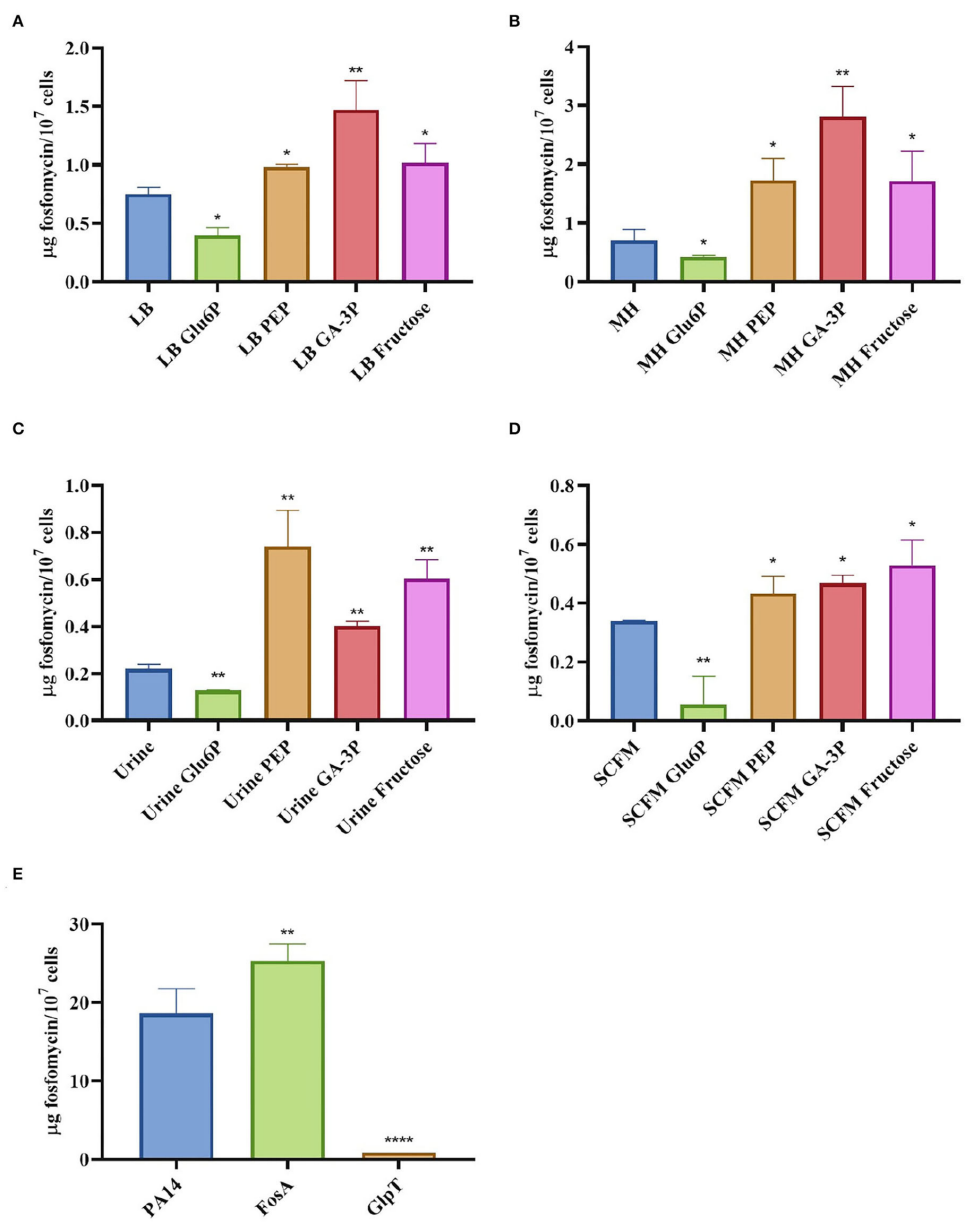
Intracellular concentration of fosfomycin in *S. maltophilia* D457 changes in the presence of different metabolites and under different growing conditions. The figure shows the fosfomycin intracellular concentration when *S. maltophilia* D457 grows under different conditions and the effect of glucose-6-phosphate (Glu6P), PEP, glyceraldehyde-3-phosphate (GA-3P), and fructose in said accumulation. As shown, there is a deficiency in the fosfomycin internalization in all media when glucose-6-phosphate is added, as well as a better internalization of the antibiotic in the presence of PEP, GA-3P, and fructose in LB **(A)**, MH **(B)**, urine **(C)**, and SCFM **(D)**. As control, the intracellular fosfomycin concentration was measured in *P. aeruginosa* PA14 wild-type strain and two isogenic mutants, one lacking the fosfomycin inactivating enzyme FosA and another one lacking the fosfomycin transporter GlpT. As shown, the absence of FosA allows higher fosfomycin accumulation, whereas the mutant lacking GlpT accumulates less antibiotic **(E)**, results that validate the reliability of the methodology. Error bars indicate standard deviations for the results from three independent replicates. Statistical significance estimated using an unpaired two-tailed *t*-test: *P < 0.01; **P < 0.001; ****P < 0.0001.

We hypothesized that the antagonistic effect of glucose-6-phosphate and the synergy of fructose, PEP, and glyceraldehyde-3-phosphate with fosfomycin should be due to changes in its intracellular concentration when the antibiotic is added in the presence of these compounds. The intracellular fosfomycin concentration was then measured under different conditions. Consistent with our hypothesis, glucose-6-phosphate reduced intracellular fosfomycin concentration, while PEP, glyceraldehyde-3-phosphate, and fructose increased such concentration, including when bacteria grow in media, such as urine or SCFM, that are close to infective conditions (Figure 2). These intracellular fosfomycin concentration changes might be due to a possible effect of the compounds tested in the regulation of the expression of the unknown fosfomycin transporters.

Breakpoints for fosfomycin have been established just for a small number of microorganisms. However, all commercial systems for measuring fosfomycin susceptibility include glucose-6-phosphate in their formulations and, as has been shown in our work, when applied to organisms, such as *S. maltophilia*, these methods are misleading, because the addition of glucose-6-phosphate underestimates fosfomycin inhibitory potential. Nevertheless, metabolites, such as fructose, PEP, or glyceraldehyde-3-phosphate, increase the activity of the antibiotic.

Besides, the distribution of inducible glucose-6-phosphate transporters in different bacteria has been studied earlier, and it has been determined that while organisms, such as *E. coli, Enterobacter aerogenes*, and *Staphylococcus aureus*, presented such transporters, other bacteria, such as *P. mirabilis, Corynebacterium diphtheriae*, and *Bacillus subtilis*, did not seem to display glucose-6-phosphate inducible internalization (Winkler, 1973). This supports that our findings could be of application for other bacteria besides *S. maltophilia*. Further studies addressing the effect of glucose-6-phosphate and other metabolites, as those here described, on fosfomycin activity might allow improving the use of fosfomycin, particularly against MDR isolates, such as *S. maltophilia* strains overexpressing SmeDEF, and implementing more realistic susceptibility tests.

## Data Availability Statement

The original contributions presented in the study are included in the article/supplementary materials, further inquiries can be directed to the corresponding author.

## Author Contributions

TG-G performed the experiments, analyzed the data and wrote the first draft of the article. JM designed the work and wrote the article. Both authors approved the last version of the manuscript.

## Funding

This work in JM Laboratory was supported by grants from the Instituto de Salud Carlos III (RD16/0016/0011), by MCIN/AEI /10.13039/501100011033 (PID2020-113521RB-I00), and from the Autonomous Community of Madrid B2017/BMD-3691. TG-G is the recipient of an FPI fellowship.

## Conflict of Interest

The authors declare that the research was conducted in the absence of any commercial or financial relationships that could be construed as a potential conflict of interest.

## Publisher's Note

All claims expressed in this article are solely those of the authors and do not necessarily represent those of their affiliated organizations, or those of the publisher, the editors and the reviewers. Any product that may be evaluated in this article, or claim that may be made by its manufacturer, is not guaranteed or endorsed by the publisher.
